# Higher Adherence to the Mediterranean Diet is Related to More Subjective Happiness in Adolescents: The Role of Health-Related Quality of Life

**DOI:** 10.3390/nu11030698

**Published:** 2019-03-25

**Authors:** Rosario Ferrer-Cascales, Natalia Albaladejo-Blázquez, Nicolás Ruiz-Robledillo, Violeta Clement-Carbonell, Miriam Sánchez-SanSegundo, Ana Zaragoza-Martí

**Affiliations:** 1 Department of Health Psychology, Faculty of Health Science, University of Alicante, 03690 Alicante, Spain; rosario.ferrer@ua.es (R.F.-C.); nicolas.ruiz@ua.es (N.R.-R.); violeta.clement@ua.es (V.C.-C.); miriam.sanchez@ua.es (M.S.-S.); 2 Department of Nursing, Faculty of Health Science, University of Alicante, 03690 Alicante, Spain; ana.zaragoza@ua.es

**Keywords:** Mediterranean diet, health-related quality of life, happiness, adolescents

## Abstract

Adherence to the Mediterranean diet (MD) has previously been related to better quality of life in the general population. Further, children and adolescents have obtained better health outcomes when they have shown high adherence to the MD in recent studies. Nevertheless, the association between the adherence to this type of diet and subjective happiness have not been previously analysed in this population. The main aim of the present study was to evaluate the relationship between MD adherence, health-related quality of life (HRQOL) and subjective happiness in a sample of 527 Spanish adolescents. Results obtained show positive associations of MD adherence with higher levels of subjective happiness and better HRQOL. Moreover, mediation analyses showed a full mediation effect of some components of HRQOL, namely, emotional well-being, mood and emotions, financial resources and social acceptance, in the association between MD adherence and subjective happiness. This study advances our understanding of the association of MD and levels of happiness in the general population, specifically in adolescents, through the positive effects of specific components of HRQOL. Future studies should evaluate other variables that could influence or also mediate this relationship, evaluating specific groups of adolescents, such as clinical samples.

## 1. Introduction

Adolescence is a period of physical growth and rapid development characterised by significant changes in cognitive, psychological and emotional domains affecting the quality of life, well-being and health status of adolescents [1]. Eating habits and nutritional knowledge acquired during adolescence are key factors for the consolidation of good dietary habits in adulthood, being protective against poor health [2,3]. Following a healthy diet during adolescence can positively affect psychological well-being and brain maturation given that most important brain structures develop rapidly during this period [4]. Therefore, improving our understanding of eating habits and lifestyle patterns of adolescents may help us identify factors that promote good health and development during adolescence.

Several epidemiological studies have already demonstrated beneficial effects of the Mediterranean diet pattern (MD) on health and psychological outcomes during adolescence [5]. This pattern is characterised by a high intake of seasonal fruit and vegetables, bread and cereals (mainly whole grain), legumes, nuts and olive oil; a moderate consumption of fish, eggs and dairy products, especially yogurt and cheeses; and an occasional consumption of meat and animal fats [6,7,8]. The MD, as a healthy eating pattern, provides an adequate amount of vitamins, minerals and antioxidants to maintain a good nutritional status, avoiding possible nutritional deficiencies and associated health problems [9,10].

Adherence to the MD has been positively related to longevity, low prevalence of chronic degenerative diseases and psychological well-being [2,11]. Specifically, in children and adolescents, good MD adherence is also associated with better academic performance and positive effects on several health problems, including asthma and allergies [3,11,12]. Hence, high MD adherence has a positive impact on health-related quality of life (HRQOL) [13,14,15], a multidimensional construct that describes physical, psychological and social well-being [16]. Although HRQOL is a marker of health and psychological functioning widely employed in clinical and general populations, few studies have evaluated the effects of eating habits on different domains of HRQOL, those that have mostly focusing on adult populations [14,15,17]. Two recent studies that have explored adherence to the MD in adolescence have found a positive association between high MD adherence and HRQOL in 456 Spanish adolescents [13,18], confirming the pattern observed in a previous study conducted with 359 Greek adolescents [11]. 

Although it has been demonstrated that high MD adherence is positively related to better HRQOL in children and adolescents, no studies have investigated the association between MD adherence and happiness in these populations. On the other hand, there has been some research into the relationship between intake patterns characteristic of MD diet, such as the consumption of fruit and vegetables, and subjective happiness [19,20,21]. Specifically, the amount of fruit and vegetables consumed was a significant predictor of higher subjective happiness scores in a recent longitudinal study conducted with 12,385 adults from a general population [20], and eating breakfast, another eating pattern recommended as part of the MD, has also been linked to higher levels of happiness [19]. Furthermore, other factors directly related to happiness, such as satisfaction with life, well-being and self-esteem, have been associated with high MD adherence in adolescents [2,13]. To our knowledge, however, the specific relationship between MD adherence and subjective happiness has not previously been studied in young people. 

In brief, few previous studies have examined the association between MD adherence and HRQOL in adolescents and, to our knowledge, none have investigated the relationship between these variables and subjective happiness in this population. To address this gap in the literature, the main aim of the present study was to evaluate the associations between the MD adherence, HRQOL and subjective happiness in a sample of Spanish adolescents. Based on previous research, we hypothesised that high MD adherence would be related to better HRQOL [11,13,18]. Although no previous studies have analysed the association between MD and subjective happiness in this population, we expected to find a positive association between these variables, given the findings of studies on specific eating patterns characteristic of MD, such as fruit and vegetable consumption [20,22].

## 2. Materials and Methods

### 2.1. Sample

Participants included 527 high school students (54.5% females; 45.5% males) ranging in age from 12 to 17 years (Mean = 14.43, Standard Deviation = 1.52) randomly selected from 5 public high schools in Alicante (Spain). 

### 2.2. Measures

#### 2.2.1. Adherence to the MD

The Mediterranean Diet Quality Index for children and teenagers (KIDMED) [10] is a questionnaire originally designed in Spain, and since employed in numerous international studies, for the evaluation of MD adherence in children and young people. Consisting of 16 questions rated on a scale ranging from 0 to 12, this tool can be self-administered or administered by an interviewer following a standard protocol. The total score on the questionnaire is classified into three levels: ≥8, indicating “optimal” MD; 4–7, improvement needed to adjust intake to Mediterranean patterns; and ≤3, very “low” diet quality. In the present study, Cronbach’s alpha for the total scale was 0.71.

#### 2.2.2. Subjective Happiness

The Subjective Happiness Scale (SHS) [23] is a self-administered four-item scale designed to provide an overall subjective judgment of happiness or unhappiness by choosing one of seven response options on a Likert scale. The Spanish version of the SHS has shown good psychometric properties, with adequate validity, reliability, and cross-cultural comparability to the English version, as well as a Cronbach’s alpha reliability of 0.81 [24]. The Cronbach’s alpha value in the current sample for this scale was 0.75.

#### 2.2.3. HRQOL

The KIDSCREEN-52 is a self-administered questionnaire measuring HRQOL in children and adolescents. It yields 10 subscales covering the following dimensions: Physical Well-Being, Psychological Well-Being, Moods and Emotions, Self-Perception, Autonomy, Parent Relations and Home Life, Financial Resources, Social Support and Peers, and School Environment and Social Acceptance. Items are scored on a 5-point Likert-type scale. The Spanish version of KIDSCREEN-52 has shown to have good psychometric properties, with adequate validity, reliability, and cross-cultural comparability [25]. In the present sample, alpha coefficients were found of 0.81 for Physical Well-Being, 0.89 for Psychological Well-Being, 0.89 for Moods and Emotions, 0.64 for Self-Perception, 0.84 for Autonomy, 0.89 for Parent Relations and Home Life, 0.87 for Financial Resources, 0.80 for Social Support and Peers, 0.82 for School Environment and 0.80 for Social Acceptance. Cronbach alpha for total HRQOL was 0.80.

### 2.3. Procedure 

The present study was a part of a large-scale study on Mediterranean diet, well-being and bullying victimisation carried out in schools in the Mediterranean city of Alicante (Spain). The study was approved by the University of Alicante (UA-2015-10-13), and parents and adolescents provided written consent to the participation in the research prior to data collection. Students between 12 and 17 years old from 5 public secondary schools who agreed to participate anonymously completed a questionnaire in a paper-and-pencil format. Inclusion criteria for the students were: (1) being present in the classroom on the day of the survey; (2) the ability to read and complete the questionnaires on their own; and (3) presenting an informed consent form signed by them and their parents allowing participation. Participants were only included in the analysis if they had completed all the questionnaires concerning the primary dependent variables assessing subjective happiness, HRQOL and MD adherence. Data were collected by a research assistant during the second and third trimester of the 2015/2016 academic year and sessions lasted 60–70 min. 

### 2.4. Statistical Analysis

Pearson’s correlations were used to analyse the relationships between MD adherence, HRQOL and subjective happiness. Hierarchical linear regression analysis was performed to determine the predictive value of MD adherence and HRQOL on subjective happiness. To confirm and clarify the association of subjective happiness with MD adherence and HRQOL, participants were divided into three groups based on their KIDMED scores (high adherence: ≥8; medium adherence: 4–7; and low adherence: ≤3) using previously reported cut-offs [10] and differences between groups were analysed employing multivariate and univariate ANOVAs. To identify specific differences between groups, Bonferroni post hoc analyses were performed. To test the mediation effect of components of HRQOL on the relationship between MD adherence and subjective happiness, the macro PROCESS for SPSS developed by Hayes was employed [26]. This macro is a path analysis modelling tool widely employed in social and health sciences for the estimation of directs and indirect effects in mediation models. This is an empirical bias-corrected bootstrapping procedure which estimates confidence intervals from repeated resampling (10,000 samples) of the observed data to test the indirect effect of MD adherence on subjective happiness through the mediation of HRQOL. Significant mediation is considered when the 95% confidence intervals did not include zero. In this case, it would be concluded that in 95% of the bootstrapped samples the effect of the MD on happiness is mediated through the included HRQOL dimensions. In small samples, bootstrapping has been shown to have benefits over traditional methods, such as linear regression or the Sobel test to test mediation effects [26]. All statistical analyses were performed using IBM SPSS Statistics for Windows, Version 24.0 (International Business Machines Corporation (IBM), Armonk, NY, USA), considering any *p* < 0.05 as significant. 

## 3. Results

### 3.1. Relationships between MD Adherence, HRQOL and Happiness 

The full pattern of correlations is summarised in Table 1. As observed, all variables evaluated were significantly positively correlated. Specifically, high MD adherence was related to high scores in subjective happiness and each HRQOL dimension (*p* < 0.001, in all cases). Similarly, a positive association was found between subjective happiness and each HRQOL dimension (*p* < 0.001, in all cases).

### 3.2. MD Adherence as a Predictor of HRQOL and Subjective Happiness

Hierarchical regression analyses were performed to analyse the role of MD adherence and HRQOL as predictors of subjective happiness, controlling for the possible effects of age and sex. In Step 1, the model included only age and sex and did not reach significance. In Step 2, MD adherence was added and the model was significant, explaining 26% of the variance in subjective happiness. In Step 3, adding all HRQOL dimensions to the model, it was significant and explained 40% of the variance in subjective happiness (Table 2).

### 3.3. Differences in HRQOL and Subjective Happiness by Level of MD Adherence

To evaluate differences in HRQOL dimensions and subjective happiness as a function of MD adherence, differences in these variables between adolescents with high, medium and low adherence to the MD were analysed controlling for age and sex. For HRQOL, significant differences were found between groups in total HRQOL, F(2,522) = 1040.208, *p* = 0.0001, η2_partial_ = 0.799 and in all its dimensions: Physical Well-Being, F(2,522) = 92.812, *p* = 0.0001, η2_partial_ = 0.262; Psychological Well-Being, F(2,522) = 248.416, *p* = 0.0001, η2_partial_ = 0.488; Moods and Emotions, F(2,522) = 272.716, *p* = 0.0001, η2_partial_ = 0.511; Self-Perception, F(2,522) = 65.624, *p* = 0.0001, η2partial = 0.201; Autonomy, F(2,522) = 195.673, *p* = 0.0001, η2_partial_ = 0.428; Parent Relations and Home Life, F(2,522) = 178.313, *p* = 0.0001, η2_partial_ = 0.406; Social Support and Peers, F(2,522) = 71.655, *p* = 0.0001, η2_partial_ = 0.215; School Environment, F(2,522) = 109.840, *p* = 0.0001, η2_partial_ = 0.296; Social Acceptance F(2,522) = 14.031, *p* = 0.0001, η2_partial_ = 0.051; and Financial Resources, F(2,522) = 41.683, *p* = 0.0001, η2_partial_ = 0.138. Age was only significant in the model in the case of Autonomy and Financial Resources, F(1,522) = 4.797, *p* = 0.029, η2_partial_ = 0.009 and F(1,522) = 6.453, *p* = 0.011, η2_partial_ = 0.012, respectively. For all variables, post hoc tests were significant, participants with high adherence to the MD obtaining higher HRQOL scores than those with medium or low adherence (*p* = 0.0001). Similarly, participants with medium adherence to MD exhibited higher levels of HRQOL than those participants with low adherence to MD (*p* = 0.0001), except in the case of Social Acceptance, in which post hoc analysis did not reveal significant differences between these groups (*p* = 0.118). With regard to subjective happiness, significant differences were found between groups, F(2,524) = 89.847, *p* = 0.0001, η2_partial_ = 0.256. Post hoc tests revealed differences between all groups, participants with high adherence to the MD obtaining higher subjective happiness scores than those with medium or low adherence (*p* = 0.0001, for all groups). In the same way, adolescents with medium adherence to MD showed higher levels of subjective happiness in comparison to adolescents with low adherence to MD (*p* = 0.0001). Means and standard deviations of HRQOL and subjective happiness scores in each group are shown in Table 3. 

### 3.4. HRQOL as a Mediator in the Association between MD Adherence and Happiness

**Figure 1:**
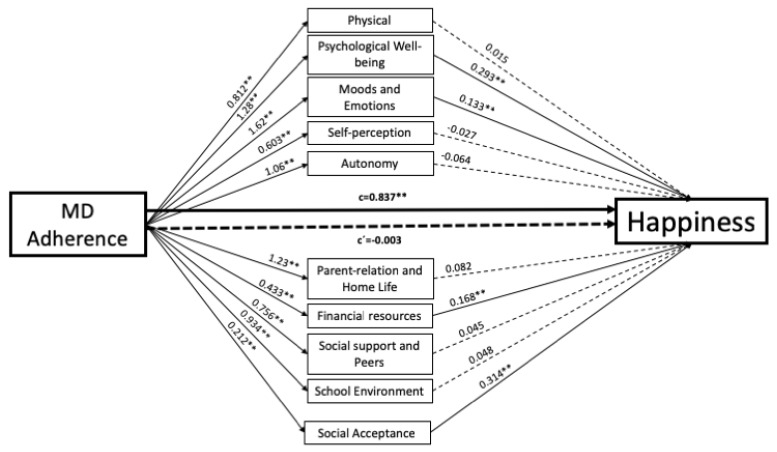
Mediation model testing the indirect association between adherence to a Mediterranean diet (MD) and happiness through the effects of each dimension of HRQOL. Dashed paths are non-significant. (** *p* < 0.01).

To evaluate possible mediation of HRQOL factors in the relationship between MD adherence and subjective happiness, we explored indirect effects using bootstrapping, controlling for possible confounders such as age and sex. Mediation analyses revealed that the total effect of MD adherence on subjective happiness was significant, B = 0.83, SE = 0.06, *p* = 0.00001. Regarding mediation effects, MD adherence, in turn, predicted scores on each of the HRQOL dimensions: Physical and Psychological Well-Being, B = 0.81, SE = 0.05, *p* = 0.00001 and B = 1.28, SE = 0.05, *p* = 0.00001 respectively; Moods and Emotions, B = 1.62, SE = 0.06, *p* = 0.00001; Self-perception, B = 0.60, SE = 0.04, *p* = 0.00001; Autonomy, B = 1.06, SE = 0.04, *p* = 0.00001; Parent Relations and Home Life, B = 1.23, SE = 0.05, *p* = 0.00001; Financial Resources, B = 0.43, SE = 0.04, *p* = 0.00001; Social Support and Peers, B = 0.75, SE = 0.05, *p* = 0.00001; School Environment, B = 0.93, SE = 0.06, *p* = 0.00001; and Social Acceptance, B = 0.21, SE = 0.03, *p* = 0.00001. Analysing the ability of the mediators to predict happiness, only the following were significant: Psychological Well-being, B = 0.29, SE = 0.05, *p* = 0.00001; Moods and Emotions, B = 0.13, SE = 0.04, *p* = 0.0045; Financial Resources, B = 0.16, SE = 0.06, *p* = 0.0073; and Social Acceptance, B = 0.31, SE = 0.07, *p* = 0.00001. The examination of the indirect effect of MD adherence on subjective happiness, through the effect of HRQOL factors, revealed a significant mediation (indirect effect = 0.84; 95% confidence interval for bias-corrected indirect effect: lower level = 0.58, upper level = 1.10). Hence, when HRQOL dimensions were included in the model as potential mediators, the association between MD adherence and subjective happiness did not reach statistical significance, B = −0.00, SE = 13, *p* = 0.9791. Overall, the final model including these mediators, F(13,513) = 28.5236, *p* = 0.00001, predicted 42% of the variance in subjective happiness (Figure 1). 

## 4. Discussion

Our results indicate that high adherence to the MD is associated with better HRQOL and higher subjective happiness in adolescents. Specifically, MD adherence was positively correlated with all of the HRQOL factors and subjective happiness. In line with this, MD adherence and HRQOL dimension scores predicted 40% of variance in subjective happiness. These results are consistent with those obtained in previous research in Spain and in other Mediterranean countries [11,13,18]. On the other hand, our study represents a significant advance in that it provides an insight into the indirect association between MD adherence on subjective happiness, testing a multiple mediation model to examine the role of HRQOL in the significant relationship between diet and happiness. 

As previously described, we found that Psychological Well-Being, Mood and Emotions, Financial Resources and Social Acceptance mediate positively in the effects of MD adherence on subjective happiness. Psychological Well-being in adolescents can be described as the positive perceptions and emotions they experience, reflecting their view of their satisfaction with life [27]. As has been previously demonstrated in the literature, higher adherence to the MD is related to the development of a healthy lifestyle which could promote a positive perception of one’s own life, and therefore, more subjective happiness [8]. This could be significantly associated with the another HRQOL dimension, Moods and Emotions, that reflects how much adolescents experience negative feelings such as loneliness, sadness, insufficiency or resignation. Higher scores in this dimension imply that the respondent rarely experiences such negative feeling and hence are indicative of good mood in adolescents [27]. 

High MD adherence entails the consumption of larger amounts of fruit and vegetables, something that has been related to greater happiness [19,20], lower incidence of depression [28,29], more life satisfaction [20] and psychological well-being [22,30,31]. Nevertheless, while the preventive mechanism of the consumption of fruit and vegetables on physical health has been well established, its effects on psychological well-being and happiness remain unclear. The effect on brain functioning of specific nutrients from the foods characteristic of the MD is a plausible explanation of the results obtained [32]. Specifically, foods included in the MD, such as fruit and vegetables, contain high levels of carbohydrates, nutrients previously associated with a high concentration of brain serotonin [22]. Further, serotonin is a neurotransmitter closely related to happiness, positive mood and motivation [33]. The production of brain serotonin is based on tryptophan, an essential amino acid precursor of serotonin found in some dairy products, dried fruit and fish, among other foods [33]. Furthermore, the formation of tryptophan is related to other types of nutrients, such as omega-3 fatty acids and minerals such as magnesium and zinc, present in fruit and vegetables, legumes and whole grains; all characteristic foods of the MD pattern [22]. Among these nutrients, omega-3 fatty acids (found in fish and nuts, seeds and dried fruit) and B-group vitamins (found in fruit and vegetables) are the most important nutrients for functioning of the central nervous system, such as neurotransmission, and for genetic expression, and in turn, adequate mood state [34]. Specifically, B-group vitamins play an important role in mitochondrial energy processes, something that could be related to feelings of vitality and happiness [31]. On the other hand, fruit and vegetables are rich in antioxidants, nutrients that protect the brain from the negative effects of oxidative stress [22]. In line with this, high levels of blood antioxidants have been associated with optimism in previous research [19,22,31]. 

Another mechanism that could be involved in the positive relationship among MD, mental health and happiness involves brain derived neurotrophic factor (BDNF) [34]. BDNF is a neurochemical related to important brain processes such as neuroplasticity and neuronal survival, and it has been associated with several mental disorders [34]. Previous research has specifically confirmed that low serum levels of BDNF are related to high levels of depression [34]. In a prospective study, MD adherence was found to be significantly associated with higher plasma levels of BDNF and a lower risk of depression [35]. Recently, researchers have turned their attention to other factors, such as the microbiome and mood [34]. Research into the positive effects of fermented foods, such as probiotics, on mental health is obtaining promising results [36]. An alternative explanation is related to self-perception of healthy food. In this regard, the consumption of certain healthy foods could be linked to an increase in positive thinking and the self-perception of developing a healthy lifestyle, leading to more happiness and well-being [37].

On the other hand, another HRQOL dimension that has been directly related to subjective happiness is Financial Resources. This dimension explores whether adolescents feel that they have enough financial resources to allow them a lifestyle which is comparable to other adolescents and provides the opportunity to do things together with peers [27]. Socioeconomic factors seem to be a one of the major determinants of adherence to the MD [38,39]. In relation to this, a previous study found that one of the main causes of low MD adherence might have been increases in the prices of some of the main components of a MD [39]. In the case of adolescents, their economic resources depend on the socioeconomic status of their parents, and this could directly influence the quality of the family diet, and, in turn, MD adherence. Furthermore, being able to have an acceptable lifestyle in which adolescents have the opportunity to participate in activities with their peers could increase social inclusion, strengthen friendships and reduce isolation, resulting in high levels of subjective happiness [40].

Finally, the last significant mediator found in our study was Social Acceptance, an indicator of not feeling bullied, feeling respected and accepted by peers [27]. On the one hand, MD adherence is associated with the maintenance of a normal weight, and this could be related to better acceptance by peers, taking into account that, traditionally, meeting the standard of beauty has been associated with better social networks, social support and social relationships during adolescence [41,42,43]. Hence, in severe cases, deviation from appearance ideals could be a significant risk factor for bullying victimisation [44,45,46]. On the other hand, MD adherence tends to go hand in hand with social sharing, such as sharing meals; and this could be related to the development of stronger social skills in adolescents and an adequate social network, such individuals being more socially prepared to interact with peers.

Although the present study represents an advance in our understanding of the relationship of subjective happiness with MD adherence and HRQOL in adolescents, certain limitations should be recognised. Firstly, it is cross-sectional research, which means that causality cannot be established. Longitudinal studies are needed to explore how MD adherence improves HRQOL in adolescents and, in turn, increases their levels of subjective happiness over time. Although previous studies employing longitudinal and experimental designs have identified a causality between the effects of specific patterns of MD and increases in well-being and happiness [20,47], new studies in this line are needed to identify the specific effects of the HRQOL on these variables. Another limitation of the study is that MD adherence, HRQOL and subjective happiness have been assessed by self-report. This means that participants may have misinterpreted questions or deliberately given incorrect answers. However, the sample size was large and the results obtained are in line with previous similar research. Future studies should evaluate which types of nutrients abundant in the MD are specifically related to HRQOL and subjective happiness in adolescents, employing instruments that enable measurement of the intake of specific nutrients. Furthermore, replication of the results obtained in clinical samples would allow us to investigate the MD as a protective factor for quality of life not only in the general population, but also in clinical samples.

## 5. Conclusions

High adherence to the MD was associated with better HRQOL and more subjective happiness in adolescents. Specifically, psychological well-being, better mood, financial resources and social acceptance were demonstrated to be significantly associated with MD adherence and happiness. Overall, the results obtained indicate the need to develop nutritional health programmes oriented to establishing adequate dietary habits in adolescents, based on the MD pattern. This could be an effective strategy for preventing several types of health problems, promoting at the same time a satisfactory lifestyle and high levels of HRQOL, resulting in high subjective happiness in adolescents. Future studies should seek to replicate the results obtained in other groups or populations, such as other age groups or clinical samples, to analyse the protective effects of MD adherence and its relationship with health status, and, at the same time, whether it helps increase or maintain subjective happiness and well-being. 

## Figures and Tables

**Table 1:** Patterns of correlations between adherence to a Mediterranean diet, happiness and health-related quality of life dimensions.

	1. Adherence to a Mediterranean Diet	2. Subjective Happiness	3. Physical Well-Being	4. Psychological Well-Being	5. Moods and Emotions	6. Self-Perception	7. Autonomy	8. Parent Relations and Home Life	9. Financial Resources	10. Social Support and Peers	11. School Environment	12. Social Acceptance	13. Total HRQoL
**1**	1	0.509 **	0.566 **	0.727 **	0.765 **	0.467 **	0.683 **	0.685 **	0.409 **	0.505 **	0.558 **	0.257 **	0.943 **
**2**		1	0.317 **	0.569 **	0.541 **	0.303 **	0.306 **	0.453 **	0.333 **	0.361 **	0.366 **	0.358 **	0.518 **
**3**			1	0.476 **	0.409 **	0.252 **	0.310 **	0.320 **	0.265 **	0.348 **	0.234 **	0.166 **	0.593 **
**4**				1	0.723 **	0.424 **	0.477 **	0.546 **	0.282 **	0.444 **	0.486 **	0.257 **	0.733 **
**5**					1	0.508 **	0.480 **	0.587 **	0.285 **	0.350 **	0.456 **	0.349 **	0.792 **
**6**						1	0.380 **	0.374 **	0.226 **	0.212 **	0.242 **	0.269 **	0.467 **
**7**							1	0.485 **	0.351 **	0.445 **	0.309 **	0.169 **	0.714 **
**8**								1	0.369 **	0.425 **	0.449 **	0.283 **	0.710 **
**9**									1	0.456 **	0.249 **	0.265 **	0.430 **
**10**										1	0.324 **	0.279 **	0.554 **
**11**											1	0.159 **	0.606 **
**12**												1	0.316 **
**13**													1

** *p* < 0.001.

**Table 2:** Predictive value of adherence to a Mediterranean diet (MD) and health-related quality of life on subjective happiness controlling for age and sex.

	Step 1			Step 2			Step 3		
	β	R^2^	ΔR^2^	β	R^2^	ΔR^2^	β	R^2^	ΔR^2^
**Age**	0.082			0.076 *			0.036		
**Sex**	0.017			−0.017			−0.015		
**F(2,526) = 1.776, *p* = 0.170**		0.003	0.007						
**Adherence to a Mediterranean diet**				0.509 ***			−0.002		
**F(3,526) = 62.831, *p* = 0.0001**					0.261	0.258 ***			
**Physical Well-being**							0.013		
**Psychological Well-being**							0.315 ***		
**Moods and Emotions**							0.173 **		
**Self-Perception**							−0.022		
**Autonomy**							−0.062		
**Parent Relations and Home Life**							0.090		
**Social Support and Peers**							0.041		
**School Environment**							0.049		
**Social Acceptance**							0.157 ***		
**Financial Resources**							0.108 **		
**F(13,513) = 28.524, *p* = 0.0001**								0.405	0.155 ***

(* *p* < 0.05; ** *p* < 0.01; *** *p* < 0.001).

**Table 3:** Subjective happiness and health-related quality of life (HRQOL) dimension scores (mean ± standard deviation) in the total sample of adolescents (*n* = 527) and in each subgroup with high (*n* = 227), medium (*n* = 197) and low (*n* = 103) adherence to a Mediterranean diet.

			MD Adherence		
		Total Sample *n* = 527	High *n* = 227	Medium *n* = 197	Low *n* = 103
	Subjective Happiness ***	21.17 ± 4.46	23.555 ± 3.28	20.21 ± 4.18	17.76 ± 4.38
**HRQOL**	Physical Well-being ***	18.05 ± 3.90	19.94 ± 3	17.13 ± 3.52	14.52 ± 3.72
	Psychological Well-being ***	23.33 ± 4.80	26.59 ± 2.61	22.57 ± 3.79	17.58 ± 4.24
	Moods and Emotions ***	26.79 ± 5.80	30.72 ± 3.31	26.08 ± 4.28	19.50 ± 5.02
	Self-Perception ***	19.49 ± 3.50	21.16 ± 2.88	18.78 ± 3.44	17.15 ± 3.05
	Autonomy ***	19.01 ± 4.24	21.74 ± 2.79	18.30 ± 3.16	14.33 ± 4.07
	Parent Relations and Home Life ***	24.66 ± 4.88	27.57 ± 2.54	24.20 ± 4.20	19.13 ± 4.94
	Social Support and Peers ***	25.05 ± 4.05	26.89 ± 2.78	24.61 ± 3.56	21.86 ± 4.98
	School Environment ***	21.39 ± 4.53	23.87 ± 3.82	20.66 ± 3.45	17.33 ± 4.38
	Social Acceptance ***	13.19 ± 2.23	13.73 ± 1.94	12.98 ± 2.16	12.43 ± 2.64
	Financial Resources ***	11.90 ± 2.86	12.89 ± 2.25	11.74 ± 2.59	10.02 ± 3.50
	Total HRQOL ***	38.52 ± 5.82	43.61 ± 2.37	37.28 ± 2.23	29.66 ± 3.57

*** *p* < 0.001.
